# Resistome and microbiome-immune interactions in an Eastern European population with high antibiotic use

**DOI:** 10.1128/spectrum.00528-26

**Published:** 2026-08-11

**Authors:** Bogdan Mirăuță, Anca-Lelia Riza, Ioana Streata, Andrei Pirvu, Stefania Dorobantu, Adina Dragos, Marius Surleac, Mihai G. Netea

**Affiliations:** 1 Human Genomics Laboratory, Functional Genomics Group, University of Medicine and Pharmacy of Craiova 121532, Craiova, Romania; 2 Romanian Bioinformatics Cluster, Târgu Mureș, Romania; 3 George Emil Palade University of Medicine, Pharmacy, Science and Technology of Târgu Mureș 37575, Târgu Mureș, Romania; 4 Department of Internal Medicine and Radboud Center for Infectious Diseases, Radboud University Medical Center 6034, Nijmegen, the Netherlands; 5 Regional Centre of Medical Genetics Dolj, County Clinical Emergency Hospital Craiova, Craiova, Romania; 6 The Research Institute of the University of Bucharest 608012, Bucharest, Romania; 7 National Institute for Infectious Diseases "Matei Balș", Bucharest, Romania; 8 Department of Immunology and Metabolism, Life & Medical Sciences Institute, University of Bonn 9374, Bonn, Germany; University of Kentucky, Lexington, Kentucky, USA

**Keywords:** antimicrobial resistance, enterobacteriaceae, gut microbiome, immunity

## Abstract

The gut microbiome influences host health, affecting gastrointestinal, metabolic, immune, cardiovascular, and neurological functions. A balanced microbiome is associated with favorable health outcomes. However, excessive antibiotic use and dietary habits can disrupt this ecosystem, leading to dysbiosis and affecting body homeostasis. This first comprehensive metagenomic analysis of the gut microbiome in a healthy Romanian cohort, a population underrepresented in microbiome studies and characterized by high antibiotic consumption, addresses a gap in current microbiome research. We report an enrichment of Enterobacteriaceae although overall composition is more comparable to other European than non-European cohorts. Community configurations align with established enterotype patterns, and our analysis provides insight into their relationship with within-phylum diversity. The analysis of antimicrobial resistance provides insight into the prevalence of resistance genes within this reservoir. We specifically report the presence of *cfr(E*), a *Clostridioides difficile* gene, and *tet(X5*), a variant from the ubiquitous *tet* family, genes not previously reported in healthy European populations. Integration with data from the European Centre for Disease Prevention and Control links the overall prevalence of resistance genes in this reservoir to antibiotic classes with higher community consumption in this population, notably beta-lactams and quinolones, highlighting potential targets for antibiotic stewardship programs. Finally, we investigate the relationship between the microbial profile and the systemic immune responses, inferred from correlations with *in vitro* cytokine production. Notably, we identify potential immune-priming roles for *Collinsella*, *Flavonifractor,* and *Bifidobacterium* species.

**IMPORTANCE:**

This first comprehensive study of the healthy gut microbiome in a Romanian cohort addresses a gap in current microbiome research, dominated by data sets from a limited number of regions. It sets a baseline for the microbiome and resistome composition of this population, and, while definitions of “healthy” microbiomes, or baseline resistomes, remain lacking, such study helps contextualize future studies and support the monitoring of dynamics. The *Enterobacteriaceae* abundance suggests a microbiome composition potentially influenced by antimicrobial consumption, a relevant pattern in a region with a high burden of nosocomial infections. In addition, the prevalence of antimicrobial resistance genes and the concordance with commonly used antibiotics in the community reinforce the need to address antibiotic use in public health strategies. Although gut microbiome–immunity relationships remain incompletely understood, our findings support a role for microbiome composition in immune-related traits and provide a valuable resource for future studies.

## INTRODUCTION

The human gut microbiome (GM) is now widely recognized as playing an important role in human health. Gut microbiome composition and diversity are influenced by multiple factors, including host genetics, age, lifestyle, diet, and medication use (1–4). Thanks to advances in sequencing technologies and analytical tools, we now have a detailed understanding of GM composition (5). Large-scale gut microbiome cohorts are now relatively common (6, 7), but they remain concentrated in a few populations (8). This limits our understanding of microbiome variation across ecological contexts and can produce population profiles with gaps, affecting both research and health policies. Previous studies on the human GM in populations from Eastern Europe such as Romania had mainly a focus on understanding disease-associated microbiome alterations (9, 10) or the enteric pathogens (11), but no comprehensive characterization of the microbiome in healthy cohorts has been performed.

Historically, antimicrobial use in Romania has been reported to exceed the European Union average as reported by ECDC, the European Centre for Disease Prevention and Control (12), for many antibiotic groups, and monitoring of antimicrobial resistance genes (ARGs) in key reservoirs has been undertaken in recent years although primarily focusing on wastewater and environmental samples (13, 14) or on the in-depth characterization of pathogen strains (15). No publicly available characterization of the ARGs in the human gut microbiome currently exists for healthy Romanian donors. The gut microbiome harbors a dense and diverse microbial community, providing a setting for both commensal and opportunistic bacteria and frequent horizontal gene transfer, making it a major reservoir of antibiotic resistance genes that can be mobilized to pathogens (16, 17). Antibiotic consumption at the population level has been linked to enrichment of ARGs in the human gut (18). However, it remains unclear whether this effect extends beyond the increased presence of *Enterobacteriaceae*, a major resistance reservoir, and evidence for specific antibiotic classes is still limited. Characterizing the gut resistome in healthy individuals can reveal baseline ARG distribution, potential dissemination pathways, and the ecological pressures shaping their dynamics.

In addition, the gut microbiome influences systemic immune responses (19), with several mechanisms being proposed: immunomodulatory effects of microbial metabolites such as short-chain fatty acids (SCFAs) or immune priming effects of certain microbial cell-wall components such as peptidoglycans and lipopolysaccharides (20). Alterations in the gut microbiome (GM) have been increasingly associated in the literature with established immune-mediated diseases. In inflammatory bowel disease, microbial co-abundance networks are disrupted (21), and changes in taxa abundance, such as *Ruminococcus*, *Blautia*, *Colidextribacter*, *Oscillospiraceae,* and *Roseburia*, have been linked to disease onset (22). *Flavonifractor plautii* was suggested to be involved in the inhibition of TNF-α expression in inflammatory environments (23) in rheumatoid arthritis (RA), and dysbiosis is associated with Th17-cell-mediated inflammation: elevated *Prevotella copri* has been observed in early rheumatoid arthritis (RA) patients (24), oral administration of *Dysosmobacter welbionis* was associated with a reduction of IL6 in the colon (25), and *Collinsella* correlates with increased inflammatory activity (26). In multiple sclerosis, patients showed reductions in *Faecalibacterium* and *Prevotella*, alongside an increase in *Actinomyces* (27).

The gut microbiome has also been associated with *in-vitro* Peripheral Blood Mononuclear Cells (PBMC) cytokine production in healthy individuals (19). Dominant patterns of microbiome variation have been proposed to underlie inter-individual differences in cytokine production capacity, with specific taxa and functional profiles correlating with distinct cytokine responses. Although the effect sizes identified to date are modest, these findings support the hypothesis that the gut microbiome may exert long-term immunomodulatory effects on the host immune system, contributing to the shaping of immune responsiveness and susceptibility to immune-mediated diseases.

In this study, we present a comprehensive analysis of the gut microbiome in a healthy Romanian cohort. We describe the overall microbiome composition, assess whether previously defined enterotypes are present in this population and their link with the community structure, and characterize the variability in microbial profiles, including comparisons with other population-level data sets. We further examine the composition and prevalence of the gut resistome. Finally, we explore the relationship between the microbiome and systemic immune responses and evaluate the cross-population consistency of these associations.

## RESULTS

### Intestinal microbiome of a Romanian population

**Fig 1:**
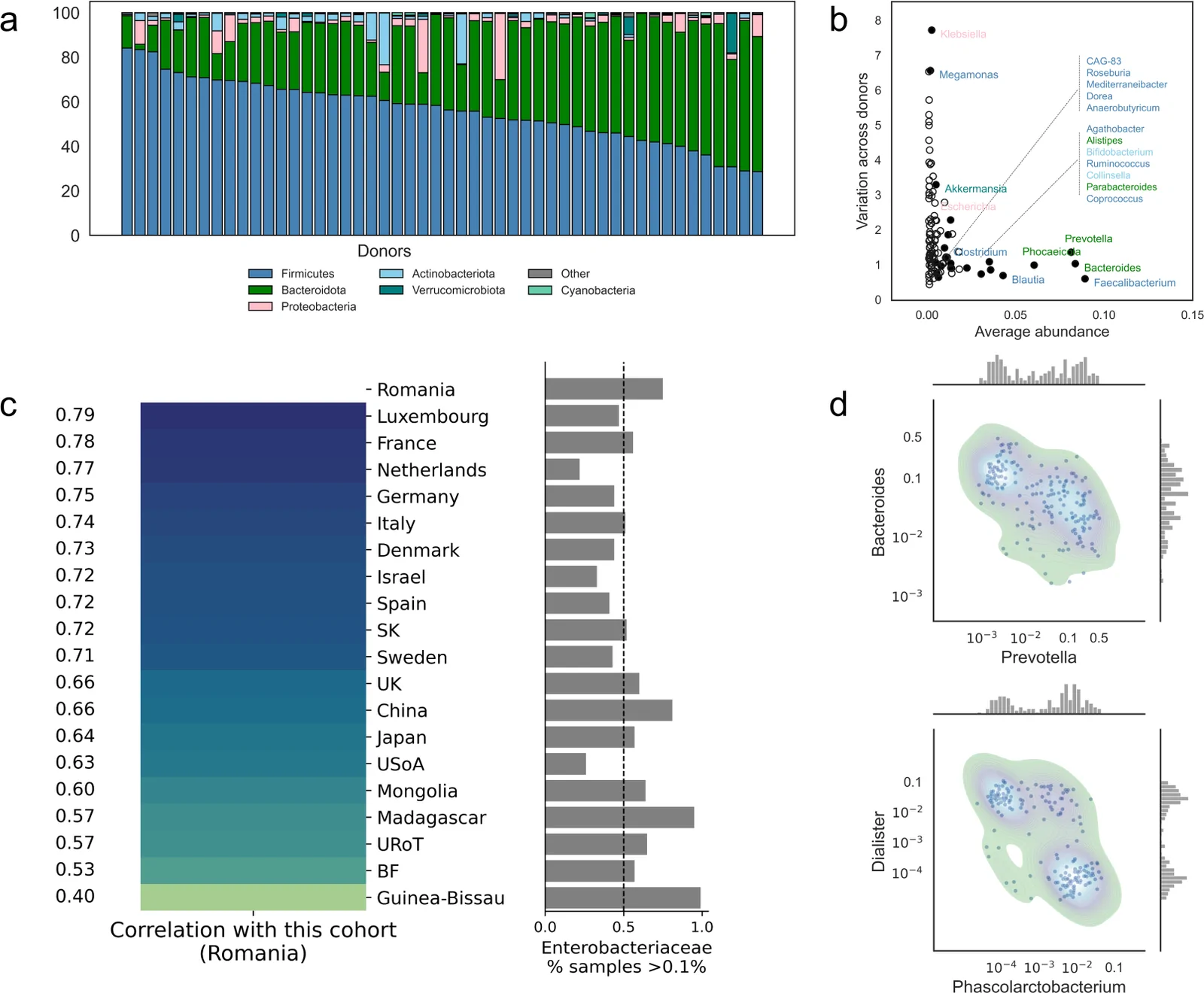
(**a**) Gut microbiome (GM) phylum composition. Samples are ordered by the relative abundance of Firmicutes (Bacillota). Fifty samples with the highest read coverage are shown. (**b**) Abundance vs variance of major bacterial genera. The *x*-axis represents the average abundance of genera (as a percentage of total mapped reads), while the *y*-axis indicates variability across samples (coefficient of variation; cv). Genera are colored by phylum according to the legend from A. We show genera detected in more than 20 samples and >0.1% average relative abundance. (**c**) Comparison with external cohorts. Heatmap of bacterial-family-level correlations, showing mean pairwise correlations across individuals. The barplot on the right shows the fraction of samples with more than 0.1% Enterobacteriaceae. (**d**) Microbiome composition types. Distribution of log abundance for the genera Bacteroides—Prevotella and Dialister—Phascolarctobacterium.

We sequenced microbiome stool samples from 215 donors, of which 184 samples with more than 30 million reads were retained for downstream analyses after QC (see Materials and Methods). The cohort had a median age of 41 years, with a bimodal distribution peaking at 25 and 50 years, and included donors from both rural and urban environments (rural: 32 female and 18 male participants; urban: 84 female and 50 male; see Table S1 at https://zenodo.org/records/20354358). The mapping to the Unified Human Gastrointestinal Genome (5) resulted in 2,915 taxa identified in at least one sample with 525 taxa detected on average in each sample (see Table S2 at https://zenodo.org/records/20354358; at minimum 0.01% relative abundance). This corresponds to 697 bacterial genera detected in at least one sample, 174 genera on average in each sample, 49 genera consistently detected (>50% of samples; >0.1% abundance), out of which 13 corresponded to uncharacterized genera from the *Clostridia* class (*Oscillospirales* and *Lachnospirales*). In 28 samples, *Prevotella* (13), *Bacteroides* (10), *Faecalibacterium* (3), *Klebsiella* (1), and *Phocaeicola* (1) genera were dominant (>25%). As expected, *Firmicutes* (*Bacillota*) and *Bacteroidetes* (*Bacteroidota*) are the dominant phyla accounting for approximately 58% and 32% of the bacterial abundance (Fig. 1a). The most abundant bacterial genera included *Faecalibacterium*, *Bacteroides*, Prevotella (*Segatella*), *Phocaeicola,* and *Blautia* (Fig. 1b).

A second mapping to Refseq, the KrakenPlusPF database (28), resulted in mapping 50% of reads to more than 14,000 taxa (see Table S3 at https://zenodo.org/records/20354358). This mapping, extended also to viruses, detected in half of the samples (>1% in 7 samples) *Carjivirus* and *Burzaovirus*, members of the order *Crassvirales* and among the most prevalent dsDNA bacteriophages of *Bacteroides* in the human gut (29).

Romanian gut microbiome composition has a better alignment with European than with non-European samples from the previous studies (30) (see Fig. 1c). Core taxa (median abundance >1%) were identified at comparable thresholds, both in this cohort and in countries with similar population characteristics (see Table S4 at https://zenodo.org/records/20354358). We observed concordance in genus-level abundances, alongside reduced abundance variation within our cohort (see Fig. S1 at https://zenodo.org/records/20354358). This includes major *Firmicutes* genera such as *Faecalibacterium* and *Blautia* (coefficient of variation <0.7). We note, however, that this lower variation may be the result of this cohort being from a geographically more homogeneous population.

This population exhibits a significant *Prevotella-Bacteroides* dichotomy, genera dominated by *Prevotella sp900557255* (24% of genera abundance) and *Bacteroides uniformis* (30%), respectively. This is consistent with the known enterotypes (31) and the expected industrial and agrarian nutritional mix in the Romanian population. We also note in this population clear succinotypes (32) with individuals having either *Phascolarctobacterium* or *Dialister* (*r* = –0.6). While *Dialister* showed multiple significant correlations with other taxa, *Phascolarctobacterium* appeared more ecologically isolated (see Table S5 at https://zenodo.org/records/20354358). Both succinate consumers show increased abundance in the *Prevotella* enterotype, a major succinate producer. The abundance of *Bacteroides* is negatively associated with alpha diversity within *Firmicutes* but positively with diversity within *Bacteroidota* and conversely for *Prevotella* (see Fig. S2 at https://zenodo.org/records/20354358; Materials and Methods). This pattern, consistent with the known effects of fiber-rich diets, metabolite production, and the more generalist role of *Bacteroides* compared to *Prevotella*, is also observed in other cohorts (see Table S6 at https://zenodo.org/records/20354358). In addition, the *Firmicutes* diversity is associated (*r* = 0.6) with CAG-170 and CAG-177 abundance, supporting recent predictions of the uncultured genus CAG-170 as a biomarker of health (33). We note that associations between phylum-level diversity and constituent taxa should be interpreted as indicating biomarker candidates rather than causal effects even though direct bacterial effects were excluded from diversity calculations (see Materials and Methods).

The *Enterobacteriaceae* family was detected constantly at higher levels than in other European countries, with 75% of samples showing more than 0.1% relative abundance, compared to 21%–56% in other European countries (Fig. 1c). We observe this family in very high abundance (>10%) in six samples, with *Klebsiella* reaching dominance (27%) in one individual. The *Escherichia* genus accounted for 75% of this family in median. This family was more prevalent in rural male donors (83% at >0.1% vs 71%–74% in other groups; 61% at >1% vs 23%–45%; see Table S7 at https://zenodo.org/records/20354358). *Enterobacteriaceae* includes many species harboring antibiotic resistance genes (ARGs), and the gut dominance of this family is associated with opportunistic infections (34). Confirming previous results (30), we observe that in our data set, species from *Enterococcaceae*, *Lactobacillaceae*, *Streptococcaceae* are co-abundant with *Enterobacteriaceae*, while *Lachnospiraceae* and *Oscillospiraceae* are negatively associated (Fisher enrichment test of associated species; pv < 0.01 for all families), enforcing the idea of the relationship between these bacteria and the *Enterobacteriaceae* colonization.

### Resistome

Antibiotic resistance genes (ARGs), biocide and metal resistance genes (BMRGs), as well as virulence factors were mapped against all metagenomic assemblies (Materials and Methods; see Table S8 at https://zenodo.org/records/20354358). To contextualize our findings, we compared them with previously published population ARG analyses (18), with data from the ECDC, and we investigated the genomic context.

**Fig 2:**
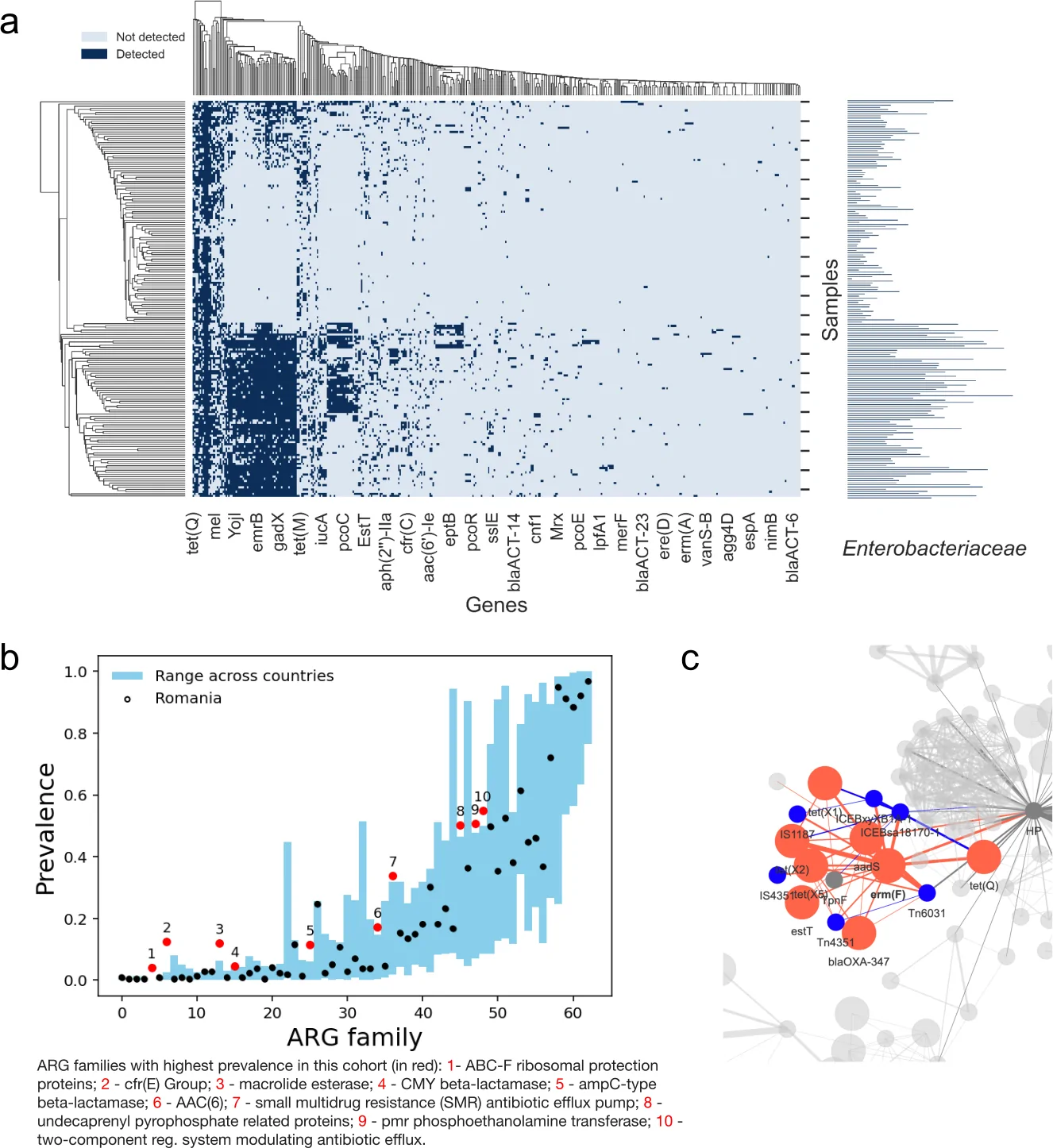
(**a**) Prevalence of antimicrobial resistance gene (ARG) families in this cohort. Left: heatmap of ARG detection in this cohort clustered by genes and samples. An arbitrary selection of gene names is shown on *x*-axis. Right: Enterobacteriaceae family abundance (log) by sample. (**b**) Prevalence of antimicrobial resistance gene (ARG) families in Romania compared with European ranges. For each ARG family (*x*-axis), the vertical blue bars represent the range of prevalence observed across countries (18; samples from Austria, Denmark, Estonia, Finland, France, Germany, Ireland, Italy, Luxembourg, Netherlands, Russia, Spain, Sweden, and the United Kingdom). Black points indicate the corresponding prevalence values observed in Romania. Red points mark ARG families where Romania’s prevalence is higher than the European range; ARG family annotations are provided under the figure. The *y*-axis shows prevalence on a scale from 0 to 1. (**c**) Co-localization of resistance determinants. Shown is a snapshot that includes the ARGs (orange) that are associated with estT, such as erm(F), blaOXA-347, tet(X5), and aadS, but also various MGEs (blue) such as the Tn6031 transposon-like element Tn6031 (blue). Edges indicate genomic proximity (<1,000 bp apart in the sequence), and the thickness indicates the number of co-occurrences. Fig. S3 at https://zenodo.org/records/20354358 expands this view to a genome-wide scale, additionally including biocide resistance genes (yellow) and phage genes (purple).

Consistent with the widespread presence of Enterobacteriaceae species in this cohort, 55% of identified resistance determinants were assigned to this family, an important reservoir of ARGs. In general, the distribution of ARG families was similar to that observed in other studies (Fig. 2a), with tetracycline-resistant ribosomal protection protein and CfxA beta-lactamase gene families being the most prevalent (>95% of samples).

Out of the 63 gene families assessed, 10 showed higher mean prevalence compared to other countries included in this analysis (see Table S9 at https://zenodo.org/records/20354358; Fig. 2b). The contigs for 6 of these 10 families were annotated to *Enterobacteriaceae*, consistent with the observed relatively high prevalence of this family in the cohort. Exceptions included aac(6’), cfr(E) group, and macrolide esterase, which were annotated to *Enterococcaceae*, *Clostridiaceae*, and *Bacteroidaceae* or *Weeksellaceae*, respectively (Blast annotation). No taxonomic assignment could be made for contigs of the miscellaneous ABC-F subfamily ATP-binding cassette ribosomal protection proteins.

We note the high (>0.1) prevalence of the *cfr(E*) gene family, originally characterized in *Clostridioides difficile* isolate DF11 (35). This gene family confers resistance to multiple antibiotics targeting the 23S rRNA, and its detection is consistent with the higher incidence of *C. difficile* infections in hospital settings in this population (36).

The macrolide esterase gene *estT*, a serine-dependent macrolide alpha/beta-hydrolase, was detected in 26 samples, and for two of these individuals, we also identified *ereD*, an erythromycin esterase protein. Of note, higher macrolide resistance was also reported by ECDC for *S. pneumoniae* isolates from the Romanian population (37) despite macrolide in the community consumption being close to the European average (38). We also note that the macrolide resistance potential of this cohort may also be suggested by the high prevalence of ABC-F subfamily genes, which mediate ribosomal protection. While the *eatAv* gene has not been directly linked to macrolide resistance, msr-type ABC-F proteins—though not enriched in our samples—have established associations with macrolide resistance (39).

*EstT* was colocalized (on the same contig) with Tn6031, a mobilizable transposon‐like element, and, in two samples, in proximity (<1,000 bp) of *aadS* or *erm(F*), also associated with macrolide resistance. We note that, in line with previous observations, *tet(X*) was found associated with Tn6031 (40). Interestingly, in seven samples, Tn6031 forms a cluster (pairwise <1,000 bp in at least one sample) with *aadS, erm(F*), *tet(X5*), and *blaOXA-347* and IS1187, IS4351 insertion sequences (Fig. 2c; see Fig. S3 at https://zenodo.org/records/20354358). The colocalization of these genes on the same MGE suggests their co-selection under combined antibiotic pressures and promotes horizontal gene transfer within microbial communities. Other colocalizations involving *bla*_OXA-347_ with macrolide (*erm*(F)) and tetracycline (*tet*(X) variants) resistance genes have previously been associated with poultry production (41).

The analysis of antibacterials for systemic use in the community (38) identifies a significantly higher than the EU/EEA mean consumptions in Romania of quinolones and beta lactams. We identify in six individuals (3% of the total) the quinolone resistance protein (qnr) family, the second highest prevalence among the countries analyzed (see Table S9 at https://zenodo.org/records/20354358).

Confirming the correlation between the beta-lactams consumption and the prevalence of beta-lactamase genes (18), we notice high values of most beta-lactamase families in our cohort at similar levels with countries of the high beta-lactam group (France, Italy, Ireland, and Spain), with notably an ubiquitous presence of CfxA. The ACI and ampC-type beta-lactamases register the highest difference compared to the European mean with 24% vs 7% and, respectively, 11% vs 3% prevalence (see Table S9 at https://zenodo.org/records/20354358). The *bla_ACI-1_* gene, an ACI beta-lactamases linked to cephalosporin resistance, appears to be subject to horizontal gene transfer (HGT) via mobile elements (42) and was previously identified in plasmids from the rumen gut microbiome (43).

The clustering analysis of resistance determinants (Materials and Methods; see Fig. S3 at https://zenodo.org/records/20354358) identified that beta-lactamases *blaEC-5/-8/-13/-15* were observed in context with the biocide resistance genes (BRG) such as *sugE; blaTEM-7* was observed to cluster with *merP/T/R* mercury resistance genes; *aph(6)-Id*, *dfrA14,* and *sul2* resistance genes associate with ICEVchBan9 (an Integrative and Conjugative Element [ICE] from *Vibrio cholerae*). While the ARGs tend to cluster together with other ARGs, MGEs, and phage genes, in this case, the BRGs usually cluster together with other BRGs only from the same functional category, such as the *arsA/B/C/D/R; soxS/R; acrE/S/F; mdtO/P/N*.

### Microbiome impact on the systemic cytokine production capacity in peripheral blood mononuclear cells

Following previous work in Western European populations (19) (hereon 500FG cohort), we investigated the relationship between the gut microbiome and the systemic host immunity in the present Eastern European cohort. To this purpose, we examined associations between changes in taxa abundances between individuals and the *in-vitro* cytokine responses of stimulated PBMCs (CS). Cytokine measurements (IL1B, TNFa, IL6, IL1Ra) were performed after PBMC stimulation with LPS, *E. coli*, *C. albicans*, *B. burgdorferi*, *S. aureus*, *M. tuberculosis*, and PHA.

We first applied a linear model to assess the contribution of GM principal components (PCs), the main drivers of variation, to the inter-individual variance in CS levels (see Materials and Methods). The Ezekiel-corrected coefficients of determination (*r*²) were within the range previously reported (up to 11%). However, the FDR-adjusted *P* values exceeded the significance threshold, indicating that overall microbiome variation is not a predictor of CS changes (see Table S10 at https://zenodo.org/records/20354358).

Next, we analyzed the associations between CS and GM abundance at species and genera resolution. We account for gender, age, BMI, and urban status effects, and we expanded our analysis to include the first five cytokine PCs (see Table S11 at https://zenodo.org/records/20354358; Materials and Methods). We identify 12 associations at species and 3 at genus level (pv < 10^−4^; 7 and, respectively, 3 at FDR 0.2).

*Collinsella* species were consistently associated with a decreased IL-6 response following bacterial and fungal stimulation. *Collinsella* has been linked to increased inflammation in disease contexts such as rheumatoid arthritis (26). The reduced cytokine production *in vitro* may reflect a distinct, context-dependent function. In a disease-free state, *Collinsella* may contribute to fine-tuning the PBMC inflammatory response, supporting immune homeostasis rather than promoting inflammation. Of note, *Collinsella aerofaciens was* identified in the 500FG cohort but was not associated with IL6 production.

*Flavonifractor plautii*, the dominant species of the *Flavonifractor* genus, was negatively associated (*r*^2^ = 0.15, pv < 10^−5^) with TNFa abundance following *S. aureus* stimulation, with similar trends observed across the other stimulation conditions. Of note, the oral administration of this species was reported to lower the expression of mRNA TNFa in epididymal adipose tissue of mice (23).

*Bifidobacterium catenulatum* showed consistent negative associations with IL6 and IL1B abundance, including a significant association with IL1B under PHA stimulation (*r*² = 0.11, pv < 10⁻⁴). Live *B. catenulatum* was previously reported to enhance *in vitro* PBMC production of IFN-gamma and was suggested to have an immunomodulatory role within the early-life gut microbiota (44).

A cross-study replication between the Dutch and Romanian cohorts was precluded by differences in analysis pipelines, our choice to account for covariates, microbiome annotation, and number of taxa identified. We note that we considered 513 species for the 500FG study, resulting in 112 species identified in both data sets, corresponding to 1,792 tested associations, out of which 26 associations being nominally significant (pv < 0.1) in both cohorts, showing partially the same effect direction (61%).

## DISCUSSION

Our data offer a snapshot into the gut microbiome of healthy individuals from an Eastern European cohort, improving understanding of microbiome variation across Europe and offering insight into a population with historically high antimicrobial drug consumption.

Comparisons with other cohorts show that our data align with gut microbiome profiles observed in other European populations. Although geographically confined, this cohort exhibits in nearly equal proportions the *Prevotella* and *Bacteroides* enterotypes. Adding to the extensive literature on diet-microbiome interactions, we find that these enterotypes associate differentially with phylum-level diversity: *Prevotella* correlates with *Firmicutes* diversity, while *Bacteroides* aligns with *Bacteroidota* diversity, patterns that are consistent across other cohorts.

There are, however, some particularities in the Romanian population that emerge from contextualizing our data through comparison with external data sets. In this context, we recognize that both protocol- and analysis-related differences may influence cross-study comparisons. Nevertheless, our use of higher-level resolutions, genus or family, mitigates the impact of methodological variations. Furthermore, the antimicrobial resistance gene analyses are supported by independent taxonomic annotation of selected ARGs using BLAST on contigs, as well as by concordant external evidence from the ECDC.

In this respect, we notice a consistent higher abundance of *Enterobacteriaceae* which likely reflects the historical antibiotic usage in this population or environmental acquisition and may indicate a gut microbiome more prone to perturbation and opportunistic colonization, potentially increasing the risk of infection (45).

The gut microbiome ARG profile from this Romanian cohort, compared with ARG data from other European populations, provides complementary insights for surveillance efforts. While resistance genes identified through gut microbiome metagenomics do not necessarily reflect phenotypic resistance at the level of individual organisms, they could indicate the overall resistance capacity of the microbial community and its potential dynamics. Within this context, we observe signals associated with increased potential resistance to quinolones, beta-lactams, and macrolides, each supported by overrepresented ARG families in our cohort and consistent with patterns reported by ECDC surveillance data. In particular, the enrichment of beta-lactamases and quinolone resistance genes aligns with reports of antimicrobial consumption in the community. This reinforces recent legislative efforts aimed at tightening community antibiotic use, suggesting that such measures are well-targeted and necessary.

We also note that the elevated resistance potential of *C. difficile* may be connected to both overall high antibiotic use and the high burden of *C. difficile infections (*CDI) in hospital settings, from where spores can be disseminated into the wider community. This is in line with our recent survey of the cause of severe infections in tertiary hospitals from the same area of South-West Romania, in which *C. difficile* was a much more frequent cause of sepsis compared to other European countries (46). As previously reported for this population (15), multidrug-resistant clones have been shown to pass from hospitals into wastewater, even after treatment. These observations highlight the need for stricter control measures.

We report the co-localization of resistance determinants, which may reflect evolutionary pressures promoting multidrug resistance. In some cases, such as the co-occurrence of *aadS*, *erm*(F), *tet*(X5), and *bla*OXA-347, this pattern suggests potential transmission from agricultural sources, specifically poultry. Existing studies on poultry AMR in Romania, e.g., (47), are limited in scope, and comprehensive metagenomic data sets are lacking, restricting our ability to determine the role of these mechanisms. Our results underscore the need for such research.

Our study provides additional support for the hypothesis that the gut microbiome influences immune function, including immune priming effects that can be observed in PBMCs *in vitro*. The global microbiome variation axes (i.e., overall community composition) did not appear to influence cytokine production capacity in PBMCs, suggesting that microbiome-related immune priming may be taxa-specific. We identified associations between cytokine responses and species belonging to the genera *Collinsella*, *Flavonifractor*, and *Bifidobacterium*, with relatively large effect sizes (*r*² ≈ 0.1) and found supporting evidence of the link of these taxa to the immune system. However, while interventional studies support a direct role for some of these taxa, the gut microbiome is an ecosystem, and associations may reflect indirect effects mediated by ecologically linked or co-abundant taxa.

The limited overlap with previously reported associations may reflect, at least in part, technical and methodological differences between studies. However, it may also indicate population-specific effects on immune signaling, which depend on prior environmental or microbial exposure. Given the high diversity of the gut microbiome (~4,000 species), the generally small effect sizes of long-term immunomodulatory interactions, and their likely population specificity, systematic characterization will require substantially larger and more harmonized cohorts (>1,000 individuals; power analysis).

This data set covers a geographically limited region, and country-wide data are needed to establish a more systematic and comprehensive map of the gut microbiome and human gut resistome in this population. Data-federation frameworks, such as those within the European life-science infrastructure (48) and the emerging national efforts of the Romanian Bioinformatics Cluster (49), could enable broader use of decentralized gut microbiome data for research, complementing health-system monitoring of resistome dynamics.

## MATERIALS AND METHODS

### Cohort

Healthy adult individuals were recruited following informed consent between May 2017 and November 2019 at the Human Genomics Laboratory, University of Medicine and Pharmacy of Craiova. We considered healthy individuals with negative medical history, under no prescribed or self-administered medication. All participants had Eastern European ancestry and were drawn from a mix of urban (Craiova) and village populations. Biological samples were collected for biobanking (serum/plasma from venous blood, urine, and stool samples) or *ex-vivo* immune experiments (PBMC isolation, stimulation followed by ELISA measurements).

### Production capacity of proinflammatory cytokines

To capture cytokine responses of immune cells, we measured the production of 24-h monocyte-derived (IL-1β, TNF-α, IL-6) after stimulation of PBMCs with eight stimuli (LPS, PHA, heat-killed *Candida albicans, Streptococcus pneumoniae, Escherichia coli, Borrelia burgdorferi, Mycobacterium tuberculosis*). Ficoll gradient isolation of PBMCs from blood was performed as previously described (50). Cells were washed twice in saline and suspended in medium (RPMI 1640) supplemented with gentamicin 10 mg/mL, L-glutamine 10 mM, and pyruvate 10 mM. PBMC stimulations were performed with 5 × 10^5^ cells/well in round-bottom 96-well plates (Corning) for 24 h at 37°C and 5% CO2. Additional details are available in the STAR Methods. Supernatants were collected and stored at −20°C until used for ELISA.

ELISA measurements were done using R&D Duoset kits, as per the manufacturers' instructions. 450 nm and 540 nm measurements were done on BMG Clariostar.

### Metagenomics analyses

Paired end (2 × 100 bp) metagenomic sequencing was performed by BGI on the DNBseq platform resulting in 5–6 G (2 × 2.5–3) per sample.

Contig assembly was performed using MetaSpades (51) within the nf-core/mag pipeline (52). Following host removal, we performed the taxonomic profiling using Kraken2 (28) within the *taxprofiler* nextflow pipeline (53).

We mapped the taxonomic lineages using GTDB (release202) (54). Of note, we used the Prevoletacea family for Prevotella, Alloprevotella, Paraprevotella, and Hallella genera (Bacteroidaceae in GTDB taxonomy). The RefSeq alignment was performed using K the KrakenPlusPF database downloaded in September 2024 from https://benlangmead.github.io/aws-indexes/k2.

Taxonomic assignments of assembled contigs were performed with Diamond (55) using the following parameters: diamond blastx -d refseq_bacteria -q [assembled contigs] -o [output file] -b 0.61 --outfmt 6 qseqid sseqid pident length mismatch staxids. For selected contigs, we annotated taxa using Blast (megablast) on the nucleotide sequences (56).

### Quality control

For ARG mapping, assembled contigs from all 215 samples were included. All other analyses were restricted to the 184 samples with sequencing depth greater than 30 million reads (median 41 million reads per sample). Association analyses were conducted after removing outlier samples identified by principal component analysis (PCA). PCA was performed on scaled microbiome and cytokine profiles, and outliers were defined as samples exceeding four standard deviations on any principal component. Principal components used in downstream association analyses were recomputed after outlier removal.

### Phylogenetic entropy

Phylogenetic entropy (57) was computed at genera level after removing the taxa whose association with diversity was being tested and by restricting the calculation to the remaining taxa of interest. Genera abundances were normalized to sum to 1 within each phylum. We used the GTDB bacterial phylogenetic trees (bac120_r226) pruned at the genus level. We followed a majority voting approach to map GTDB nodes to the taxa names used in this study.

### ARG methods

The metagenomics assemblies (contigs) have been subject of antibiotic resistance genes (ARG) predictions using CARD (58), ResFinder (59), and BacAnt (60). Diamond software was used to compare the contig sequences against the protein databases by BlastX (the following command was used: diamond blastx --max-target-seqs 0 --more-sensitive --id 70 -p 8 --subject-cover 90) (55). The resulting set has at least 70% identity and 90% coverage on the reference sequences. The analysis of the presented results was performed only on the subset having at least 90% to 100% identity. R was used for graph representation. The genomic context clustering has been analyzed with an in-house script that looks at genes that are in close proximity in the genome (<1,000 bp) or in the contig where the predicted genes have been found. The context clustering graph has been generated in R based on the subsets that contained at least two antibiotic or biocide resistance genes in proximity with one another.

Between countries analysis was performed at ARG class resolution. We mapped ARG (CARD ARO Ids) to ARG class by retrieving the parent term in the CARD ontology 4.0.1 (aro.json and aro.obo files). We used prevalence (detection) of ARG or ARG class and averaged the prevalence across the samples in a country.

### Explained variance

We ran a linear model with the first 20 PCs and calculated the Ezekiel r2 estimator, 1−(*N*−1)/(*N*−*p*−1)×(1−*r*^2^), where *N* is the number of samples and *p* is the number of explanatory variables (PCs in this case). *P* values were computed using a permutation strategy keeping the PCs unchanged and permuting the cytokine profiles. We then followed a Benjamini-Hochberg strategy to account for multiple testing.

### Microbiome associations with cytokine abundance

We estimated the relationship between bacterial and cytokine abundances by calculating Pearson correlations on rank-transformed data on 131 donors for which cytokine, microbiome, and metadata were available. We adjusted for gender, age, urban status, and BMI. Age was categorized into two groups (18–39 and 39–65 years; *n* = 66 and, respectively, *n* = 65), and samples from individuals older than 65 years (*n* = 7) were excluded. We also excluded two samples with BMI >35 and categorized BMI into two groups (under and over 25; *n* = 70 and, respectively, *n* = 61). Correlations were computed on ranked data after regressing out the covariates using linear regression. We considered bacterial species with average relative abundance above 0.001% and detected in at least 10 samples >0.01%.

## Data Availability

The raw microbiome sequencing data generated in this study are publicly available in the European Nucleotide Archive (ENA) under accession number ERP194162. Metagenome-assembled contigs corresponding to antimicrobial resistance genes, together with all referenced supplementary tables, are publicly available through Zenodo (https://zenodo.org/records/20354358). The cytokine data can be requested from the corresponding author.
